# Accessibility to primary health care in Belgium: an evaluation of policies awarding financial assistance in shortage areas

**DOI:** 10.1186/1471-2296-14-122

**Published:** 2013-08-22

**Authors:** Bart Dewulf, Tijs Neutens, Yves De Weerdt, Nico Van de Weghe

**Affiliations:** 1 Department of Geography, Ghent University, Krijgslaan 281, S8, B-9000 Ghent, Belgium; 2 Research Foundation Flanders, Egmontstraat 5, B-1000 Brussels, Belgium; 3 VITO, Boeretang 200, Mol B-2400, Belgium

**Keywords:** Primary health care, Accessibility, Geographical information systems (GIS), Physician-to-population ratio (PPR), Floating catchment area (FCA)

## Abstract

**Background:**

In many countries, financial assistance is awarded to physicians who settle in an area that is designated as a shortage area to prevent unequal accessibility to primary health care. Today, however, policy makers use fairly simple methods to define health care accessibility, with physician-to-population ratios (PPRs) within predefined administrative boundaries being overwhelmingly favoured. Our purpose is to verify whether these simple methods are accurate enough for adequately designating medical shortage areas and explore how these perform relative to more advanced GIS-based methods.

**Methods:**

Using a geographical information system (GIS), we conduct a nation-wide study of accessibility to primary care physicians in Belgium using four different methods: PPR, distance to closest physician, cumulative opportunity, and floating catchment area (FCA) methods.

**Results:**

The official method used by policy makers in Belgium (calculating PPR per physician zone) offers only a crude representation of health care accessibility, especially because large contiguous areas (physician zones) are considered. We found substantial differences in the number and spatial distribution of medical shortage areas when applying different methods.

**Conclusions:**

The assessment of spatial health care accessibility and concomitant policy initiatives are affected by and dependent on the methodology used. The major disadvantage of PPR methods is its aggregated approach, masking subtle local variations. Some simple GIS methods overcome this issue, but have limitations in terms of conceptualisation of physician interaction and distance decay. Conceptually, the enhanced 2-step floating catchment area (E2SFCA) method, an advanced FCA method, was found to be most appropriate for supporting areal health care policies, since this method is able to calculate accessibility at a small scale (e.g. census tracts), takes interaction between physicians into account, and considers distance decay. While at present in health care research methodological differences and modifiable areal unit problems have remained largely overlooked, this manuscript shows that these aspects have a significant influence on the insights obtained. Hence, it is important for policy makers to ascertain to what extent their policy evaluations hold under different scales of analysis and when different methods are used.

## Introduction

Primary health care is the first line of defence for a population and can prevent or reduce unnecessary, expensive speciality care [1-3]. Hence, accessibility to primary health care is considered a fundamental right and an important facilitator of overall population health.

Ensuring equal accessibility to primary care for those in equal need has long been of concern to public health policy makers, service providers, researchers, and consumers alike. Various countries have implemented incentive health programs to redress spatial gaps in service provision. In the US, for instance, the federal government spends over one billion dollars a year on programs (e.g. National Health Service Corps Program) that seek to improve accessibility to health care by, among others, offering financial support to health care professionals, who serve shortage areas [3,4]. Likewise, in Belgium, the Rijksinstituut voor Ziekte- en Invaliditeitsverzekering (RIZIV; *‘National Institute for Disease and Invalidity Insurance’*) has an incentive program, called Impulseo I, which awards 20,000 euros to physicians who settle in a physician zone (consisting of multiple municipalities) with a low physician-to-population ratio – that is, less than 90 physicians/100,000 inhabitants, or both less than 120 physicians/100,000 inhabitants and less than 125 inhabitants/km^2^[5].

While medical deficits determined on the basis of zonal physician-to-population ratios can be derived easily from a simple spread sheet, they may – if not complemented by a more in-depth spatial analysis – generate only crude and even misleading insights into the health provision landscape. Such a spatial analysis can be achieved by using geographical information systems (GIS) that enable to input, store, manipulate, analyse, and visualise spatial information [6]. The analytical power of GIS holds tremendous value for public health reformers in uncovering and mapping socio-spatial disparities in health care accessibility, and monitoring the impact of policy initiatives aimed at reducing these [7,8]. However, it is regrettable to observe with Joyce [9] that “despite GIS having applications in fields as diverse as engineering and anthropology, the potential of GIS has yet to be fully exploited in health settings”. By and large, policy decisions in Belgium and elsewhere are based on rather crude definitions of what constitutes accessibility, disregarding the full diversity of sophisticated indicators that have been proposed in the academic literature.

In this paper, we will examine the validity of the Belgian policy directives regarding financial support for physicians using different GIS-based methods to designate underserved areas of primary health care. The general aim is to evaluate to what extent spatial health care accessibility and concomitant policy initiatives are affected by and depend on the method and scale of analysis used. This general aim unfolds into two specific objectives. The first objective is to statistically analyse the results from four different GIS methods using cross tabs and compare these with current practice in Belgium. The second objective is to perform an analysis of the spatial distribution of shortage areas.

## Background

Health care accessibility can be classified into two categories: revealed accessibility and potential accessibility [10-12]. The former deals with the actual use of health care services, while the latter focuses on the aggregated supply of available health care in an area and thus the potential use of services. Both can be further subdivided into spatial and non-spatial accessibility. Spatial accessibility is based on spatial factors, including the distribution of primary health care providers (supply; in Belgium mostly self-employed physicians) and population (demand), and the distance/time between supply and demand [13]. Non-spatial accessibility is based on non-spatial factors such as socio-economic factors, the health status of the population, and people’s knowledge about the health care system [10,13]. It is essential toward any effective government intervention program to identify where potential shortage areas are located [2,14]. In this paper, we will focus on potential spatial accessibility (henceforth briefly referred to as accessibility).

To calculate primary health care accessibility in general and physician shortage areas in particular, various methods can be used. Simple methods include distance/time (Euclidean, Manhattan, or network) to the nearest physician, the average distance/time to a certain number of physicians, and cumulative opportunity (which is calculated as the number of physicians within a certain distance/time) [15,16]. However, these methods give only a rough estimation of accessibility. Distance to the nearest provider for example does not capture full accessibility, because it is often observed that people bypass the nearest service when there is more than one service to choose from [17-21]. Cumulative opportunity does not take interaction between population and physicians, and competition between physicians into consideration [18,21].

Physicians co-exist in a network of overlapping catchments, and people are free to choose health care wherever and from whomever. Therefore, physicians compete for the population’s use of their services [21]. Some methods are based on PPRs to measure accessibility in a predefined area, as is the case in Impulseo I. The advantage of these methods is that they are easy to implement (no GIS tools needed) and comprehend. In spite of this, traditional PPRs have several limitations [22-24]. First of all, PPRs are usually calculated with zonal data, which are based on administrative boundaries (e.g. municipalities). In Impulseo I, PPRs are calculated per physician zone, which have a median area of 86.53 km^2^ with a median population of 36,613. When using administrative zones boundaries are considered impermeable and as a result, the interaction across borders is not sufficiently taken into consideration [10,14]. Second, the physical separation with physicians is not equal for all inhabitants residing in the same zone, which causes accessibility to vary within that zone [14,25]. Nonetheless, the PPR method assumes equal accessibility to services irrespective of where individuals live within the zone [6]. Calculating PPRs within administrative borders can hence strongly influence the results when working on a different scale level, which constitutes a well-known source of statistical bias in geography termed the modifiable areal unit problem (MAUP) [26]. MAUP generally occurs when point-based measures of spatial phenomena are aggregated into districts.

A method that partly overcomes both limitations is the 2-step floating catchment area (2SFCA) method, developed by Luo & Wang [27] and based on the spatial decomposition idea by Radke & Mu [28]. In this method, a circle (catchment) of some reasonable radius (matched on the road network) centred on the census tract centroid is used as the basic unit instead of using a predefined administrative boundary to calculate PPRs.

**Figure 1:**
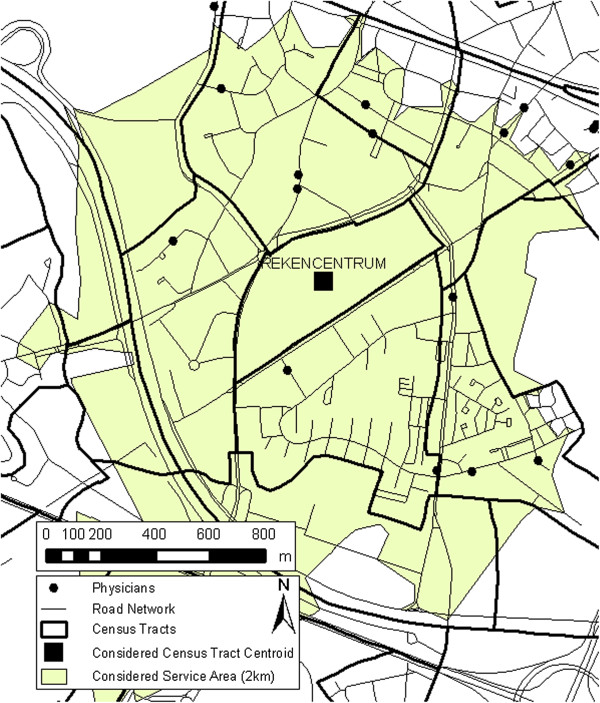
Example of a service area around a census tract centroid, which shows the alignment with the road network and the intersection with the census tract boundaries.

Because catchments are used instead of administrative borders, crossing of borders is now possible. This can be seen in Figure 1, where an example of a catchment from a centroid of a census tract (in casu ‘Rekencentrum’ in Ghent) is shown. This catchment is strongly related with the road network and intersects with the census tract boundaries. The catchment radius is defined as the maximum distance/time along the road network, where all physicians are deemed accessible and equally proximate to that particular population (centered at the census tract centroid). The catchment that is hereby formed floats from census tract centroid to census tract centroid, hence the name of the method. This way, shortage areas with PPRs lower than a predefined value can be defined.

The PPR per census tract centroid is calculated in two steps. In the first step, the PPR is first calculated on each physician location, using formula (1). In the second step, the PPR is calculated per census tract centroid by averaging all PPRs from step one, using formula (2). Doing so, the method considers interaction between population and physicians [6,21].

(1) $$R_{j}=\frac{S_{j}}{\sum_{k\in \left\{d_{\mathrm{kj}}\leq d_{o}\right\}}P_{k}},$$

(2) $$A_{i}=\sum {}_{j}R_{j},$$

where *R*_*J*_ is the PPR at physician location *j*, *S*_*j*_ is the number of physicians at location *j*, *P*_*k*_ is the population of census tract *k* whose centroid falls within the physician catchments (that is, *d*_*kj*_ ≤ *d*_0_), *d*_*kj*_ is the travel distance between *k* and *j*, *d*_0_ is the travel distance radius of the catchment, and *A*_*i*_ represents the accessibility at census tract *i* to physicians.

In the 2SFCA method, the assumption of equal accessibility within the catchment and no accessibility outside stands [24,29]. The enhanced 2-step floating catchment area (E2SFCA) method overcomes this by applying a distance decay function [3]. Each catchment is divided into multiple sub catchments, which receive varying weights defined by a weight function, which can be adjusted depending on the type or importance of a service. Formulas (1) and (2) are hereby transformed into formulas (3) and (4). By doing this, it is accepted that services that are closer to the census tract centroid are more accessible. The use of this function is required when working across large geographies, which is often the case for health policies at national level [3].

(3) $$R_{j}=\frac{S_{j}}{\sum_{k\in \left\{d_{\mathrm{kj}}\leq D_{r}\right\}}P_{k}W_{r}},$$

(4) $$A_{i}=\sum {}_{j}R_{j}W_{r},$$

where *W*_*r*_ is the distance weight for the *r*th travel time zone defined by the distance decay weight function capturing the distance decay of accessibility to physician *j*.

This E2SFCA method is now considered the standard FCA method, and is used in a variety of studies [3,30,31]. McGrail [21] suggests to use a variable catchment size function, depending on the population type (urban or rural) and service. The reason for this is that rural populations are generally more accustomed to travel further to a service location, and urban populations will mostly access services in a closer proximity because service locations are densely located. However, since in Belgium differences between urban and rural populations are not as big as in, say, Australia or North America, such function will not be applied here.

FCA-based methods have the advantage of calculating accessibility on a much smaller scale than is feasible with traditional PPRs [24].

## Methods

**Figure 2:**
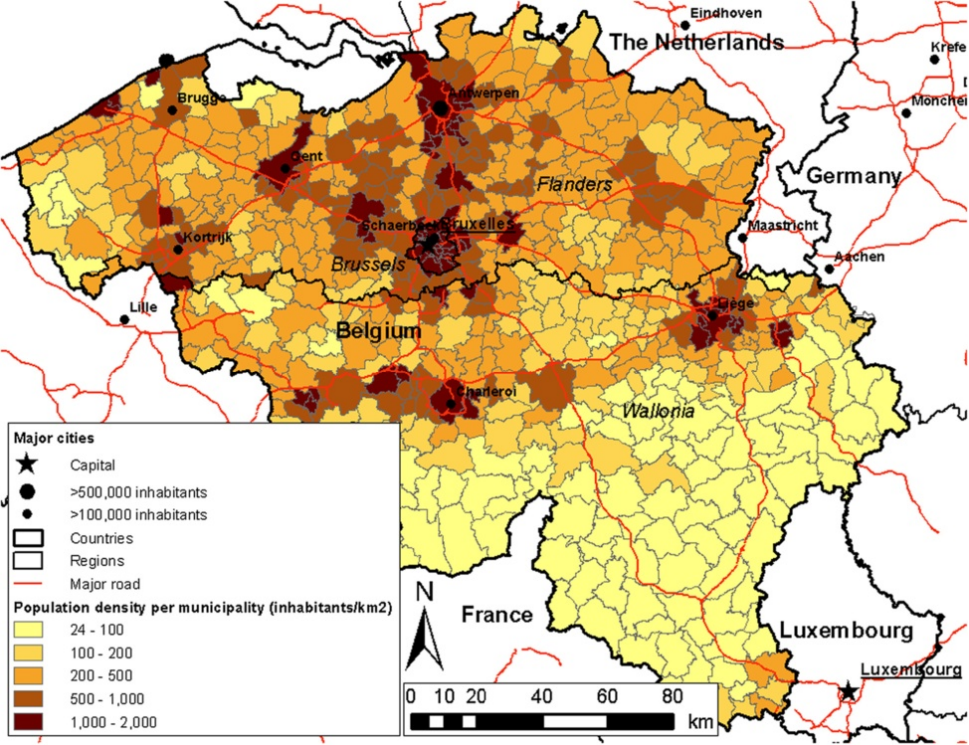
Study area indicating Belgium and its neighbouring countries, the division of Belgium in three regions (Flanders, Wallonia, and Brussels), the major motorways, and the population density per municipality.

The study area of the paper is the whole country of Belgium (see Figure 2), with a population of approximately 10.8 million inhabitants on an area of 30,528 km^2^. Belgium is divided into 161 physician zones (median area: 86.53 km^2^, median population: 36,613), 589 municipalities (median area: 40.10 km^2^, median population: 11,702), and 19,781 census tracts (median area: 0.51 km^2^, median population: 310). A physician zone collects physicians who are active in a contiguous geographic area that consists of one or more municipalities, or is part of a municipality in the large agglomerations of Antwerp, Brussels, Ghent, and Liège. Population data per census tract of the year 2011 were used, together with the geocoded addresses of all active physicians (in total, 10,353) in Belgium in that same year. Physicians are considered active when they have at least 500 patient contacts per year, which is concurrent with the official definition of Impulseo I. In order to calculate shortest paths between physicians and census tracts centroids, and subsequently define service areas we have used a transportation network shapefile (TeleAtlas MultiNet®), consisting of a detailed topological representation of the Belgian road network.

All calculations were performed in ArcGIS 9.3™. Four types of methods to measure accessibility were selected on the basis of their frequent use in health studies: (i) PPR, (ii) distance to closest physician(s), (iii) cumulative opportunity, and (iv) floating catchment area (FCA) methods. To explore the scale effect of the MAUP, different spatial units of analysis were used: physician zone, municipality, and census tract.

First, PPRs were calculated per physician zone (which is also done in the official Impulseo I method) and per municipality. PPRs have not been calculated per census tract, simply because a lot of census tracts contain no physicians (which would yield a PPR of zero), while in fact there is a physician located in its proximity (e.g. in one of the adjacent tracts). This is often referred to as the small population problem.

Second, simple GIS methods expressing physical separation between population and physicians were calculated per census tract, including distance to the nearest physician, and mean distance to the nearest three physicians. This last method was included, because people often bypass the nearest physician [18,20].

Third, cumulative opportunity was calculated per census tract as the number of physicians within a certain distance from its centroid. These thresholds are often arbitrary and difficult to select. Based on previous studies [6,16], we have used buffers of 5 and 10 km.

Finally, two types of FCA-based methods were computed per census tract. It is noted that calculating FCA measures per census tract is meaningful because in FCA methods, crossing administrative borders, including those with zero physicians, is possible [16]. This spatial smoothing effect thus solves the small population problem [32]. Based on prior work [6,16] and in analogy with the cumulative opportunity metric, the 2SFCA was performed with a catchment of 5 and 10 km. Following McGrail [21], in the E2SFCA, we used the following slow step-decay function: 1, 0.80, 0.55, and 0.15, respectively for the catchments 1 km, 2 km, 5 km, and 10 km. A slow step-decay function is preferred to a fast step-decay function, because in the context of Belgium physicians located outside the 1 km catchment not necessarily have a low accessibility.

To implement the accessibility measures above, distances and car travel times were calculated along the street network. This was done by using the Network Analyst Extension of ArcGIS 9.3™. However, like Apparicio [16], we found a strong positive correlation between both the shortest network distance and car travel time (two-tailed Pearson *r* = 0.949, *p* < 0.001), and therefore in this paper we only elucidate the results using network distances. Also, network distances are preferred because we did not want to presuppose the transport mode used to get to a physician by using mode-specific speeds for calculating travel time [2,16,33].

Impulseo I defines the following criteria to determine whether an area is underserved: (i) PPR <90 physicians/100,000 inhabitants, or (ii) <120 physicians/100,000 inhabitants and population density <125 inhabitants/km^2^. For the FCA based methods (2SFCA, and E2SFCA), we have used the same criteria, but without criterion (ii). This is because population density is already indirectly incorporated in the FCA methods as it accounts for the fact that people compete for physicians (and vice versa). For average distance to the (three) closest physician(s) and cumulative opportunity within 5 and 10 km, the same number of census tracts as resulting from the official Impulseo I method (i.e. PPR per physician zone) have been designated as shortage area. This means that a threshold distance and cumulative opportunity value had to be set, with all census tracts having an accessibility value above/below this threshold being designated as underserved.

The different methods will be tested on correlation using a two-tailed Pearson test in SPSS Statistics 21™. The methods that did not exhibit high mutual correlation will then be compared with each other and with the official Impulseo I method using a large cross tab and by visualising the spatial data in maps. To accomplish the second objective, i.e. the detailed spatial analysis of the conceptually most advanced method (E2SFCA method), a geographical analysis will be performed.

## Results

### Statistical analysis

Table 1 shows the results from a two-tailed Pearson correlation test, indicating the correlation coefficient (colour coded) and its significance. It can be observed that there is a strong and significant correlation (0.739) between the distance methods (Dist1 and Dist3). In addition, there is a strong correlation (0.653) between the cumulative opportunity methods (Cum5 and Cum10). A moderate to strong correlation is noted among the different FCA methods (2SFCA5, 2SFCA10, and E2SFCA). The E2SFCA method in particular has a rather strong correlation with the other FCA-based methods. It should also be noted that the correlation between different methods is rather weak (mostly lower than 0.4). Based on the outcome of this correlation analysis, we have selected four specific methods (one per method group) for further analysis: PPR per municipality (PPR_Mun), distance to three closest physicians (Dist3), cumulative opportunity within 10 km (Cum10), and the E2SFCA method.

**Table 1:** Results from the two-tailed Pearson correlation test

***Method***	***PPR_Phys***	***PPR_Mun***	***Dist1***	***Dist3***	***Cum5***	***Cum10***	***2SFCA5***	***2SFCA10***	***E2SFCA***
***PPR_Phys***	1*								
***PPR_Mun***	0.396*	1*							
***Dist1***	0.176*	0.110*	1*						
***Dist3***	0.203*	0.122*	0.739*	1*					
***Cum5***	0.269*	0.152*	0.410*	0.543*	1*				
***Cum10***	0.321*	0.121*	0.355*	0.457*	0.653*	1*			
***2SFCA5***	0.169*	0.277*	0.201*	0.218*	0.244*	0.045*	1*		
***2SFCA10***	0.207*	0.215*	0.149*	0.155*	0.145*	0.190*	0.199*	1*	
***E2SFCA***	0.192*	0.267*	0.341*	0.367*	0.310*	0.131*	0.597*	0.488*	1*

* Correlation is significant at the 0.01 level.

The results of these methods will be compared mutually as well as against the official Impulseo I method (that is PPR per physician zone; PPR_Phys) using a cross tab (Table 2), showing the number (*Count*) and percentage (*Table %*) of underserved census tracts per method.

**Table 2:** Cross tab showing the comparison between the official Impulseo I method and the four selected methods

			**PPR_Phys**		**PPR_Mun**		**Dist3**		**Cum10**		**E2SFCA**	
			*No shortage area*	*Shortage area*	*No shortage area*	*Shortage area*	*No shortage area*	*Shortage area*	*No shortage area*	*Shortage area*	*No shortage area*	*Shortage area*
**PPR_Phys**	*No shortage area*	Count	11,624	0								
		Table %	58.8%	0%								
	*Shortage area*	Count	0	8,157								
		Table %	0%	41.2%								
**PPR_Mun**	*No shortage area*	Count	7,967	2,316	10,283	0						
		Table %	40.3%	11.7%	52,0%	0%						
	*Shortage area*	Count	3,657	5,841	0	9,498						
		Table %	18.5%	29.5%	0%	48,0%						
**Dist3**	*No shortage area*	Count	7,802	3,822	6,635	4,989	11,624	0				
		Table %	39.4%	19.3%	33.5%	25.2%	58.8%	0%				
	*Shortage area*	Count	3,822	4,335	3,648	4,509	0	8,157				
		Table %	19.3%	21.9%	18.4%	22.8%	0%	41.2%				
**Cum10**	*No shortage area*	Count	8,337	3,229	6,601	4,965	8,991	2,575	11,566	0		
		Table %	42.1%	16.3%	33.4%	25.1%	45.5%	13.0%	58.5%	0%		
	*Shortage area*	Count	3,287	4,928	3,682	4,533	2,633	5,582	0	8,215		
		Table %	16.6%	24.9%	18.6%	22.9%	13.3%	28.2%	0%	41.5%		
**E2SFCA**	*No shortage area*	Count	7,285	3,528	6,935	3,878	8,135	2,678	6,957	3,856	10,813	0
		Table %	36.8%	17.8%	35.1%	19.6%	41.1%	13.5%	35.2%	19.5%	54.7%	0%
	*Shortage area*	Count	4,339	4,629	3,348	5,62	3,489	5,479	4,609	4,359	0	8,968
		Table %	21.9%	23.4%	16.9%	28.4%	17.6%	27.7%	23.3%	22,0%	0%	45.3%

Table 2 shows that in total 8,157 census tracts (41.2% of all census tracts) are underserved and should thus receive financial assistance, when using the official Impulseo I method (PPR_Phys). In contrast, when using the first selected method (PPR_Mun) 9,498 census tracts (48.0%) are identified as shortage areas (Table 2). In total, 5,841 census tracts (29.5%, compared to 41.2% from the official method) are in both methods consistently classified as underserved, while 7,967 census tracts (40.3%, compared to 58.8% from the official method) are in both methods consistently not identified as shortage areas. This PPR_Mun method is most similar to the official PPR_Phys method, simply because both are based on calculating PPRs.

The second alternative method (Dist3) consists of calculating the average distance to the three closest physicians. The average value of all average distances from each census tract centroid to the closest three physicians for the whole of Belgium is 2,045 m. In order to identify the same amount (8,157) of underserved census tracts as in the official Impulseo I method, a threshold value (1,878 m) was determined so that exactly 8,157 census tracts had a value higher than this threshold and were thus classified as underserved. Table 2 shows that 4,335 census tracts (21.9%/41.2%) are shortage areas in both methods (Dist3 and PPR_Phys). It can also be deduced that 61.3% (39.4% + 21.9%) of all census tracts were in both the official PPR_Phys and the Dist3 method classified consistently, while in 38.6% (19.3% + 19.3%) of all census tracts there are inconsistent evaluations as to whether or not financial assistance should be awarded.

For the third method (Cum10), we calculated the number of physicians within 10 km for all census tracts, and considered the 8,157 census tracts with the lowest number of physicians within 10 km. We found 8,215 census tracts with less than 58 physicians within 10 km. Following Table 2, 4,928 census tracts (24.9%/41.2%) are identified as shortage areas in both methods (official and Cum10 method). 67.0% (42.1% + 24.9%) of all census tracts are assessed consistently in both methods, while 32.9%/58.8% (16.6% + 16.3%) are different.

Finally, when using the E2SFCA method, 8,968 census tracts (45.3%) are considered underserved and 10,813 (54.7%) are not (see Table 2). In 4,629 census tracts (23.4%/41.2%) financial assistance is awarded in both methods (official and E2SFCA method) and in 7,285 (36.8%/58.8%) not. Also, 60.2% (36.8% + 23.4%) of all census tracts are equally identified in both methods (official and E2SFCA method) and 39.7% (21.9% + 17.8%) are different.

**Figure 3:**
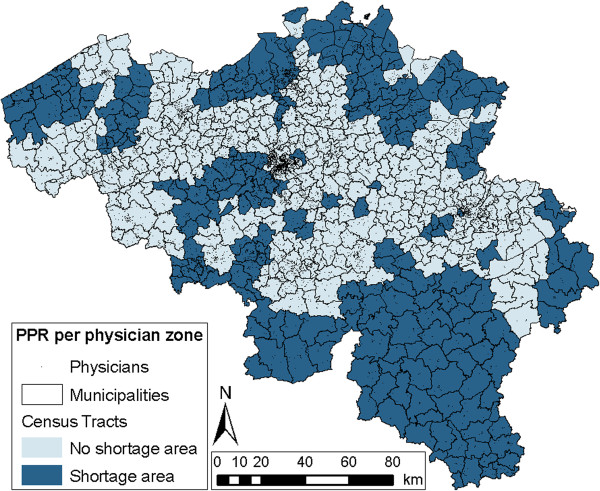
Map showing which census tracts are considered shortage areas, using the official Impulseo I method (PPR per physician zone), additionally indicating the location of all physicians.

**Figure 4:**
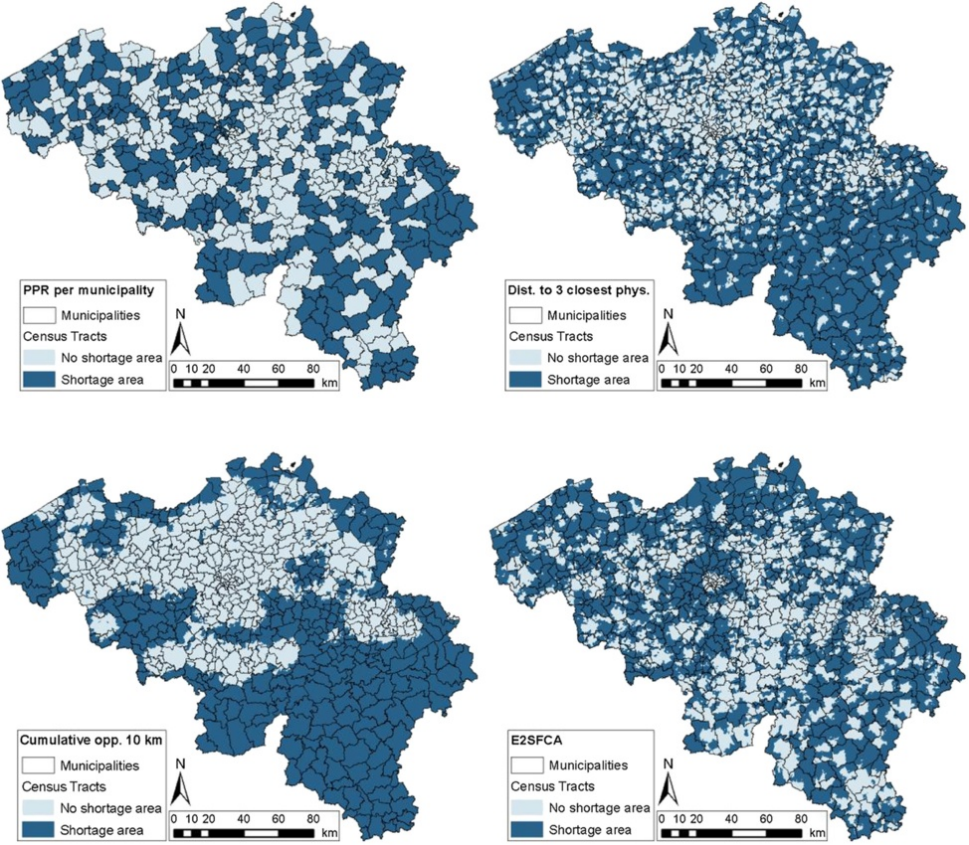
Map showing which census tracts are considered underserved, using the (i) PPR per municipality, (ii) distance to three closest physicians, (iii) cumulative opportunity within 10 km, and (iv) E2SFCA method.

The results of the four methods can also be visually represented in maps. Figures 3 and 4 show the spatial distribution of census tracts that are considered as shortage areas in Belgium, for the official Impulseo I method as well as the four selected methods. Additionally, Table 3 provides some general numbers for each of these methods. The table summarizes the percentage of census tracts that are underserved, but also shows the percentage of underserved area and population. In order to illustrate the potential financial implications of methodological choices, the last column indicates the money that would have to be awarded per year assuming an increase of one physician per 10 underserved census tracts per year.

**Table 3:** Percentage of underserved census tracts, area, and population, and amount of money needed for the official and the four selected methods

***Method***	**Census tracts underserved (%)**	**Area underserved (%)**	**Population underserved (%)**	**Amount of money needed per year**^**a **^**(€)**
**PPR_Phys**	41.2	51.9	35.3	16,314,000
**PPR_Mun**	48.0	51.2	47.7	18,996,000
**Dist3**	41.2	66.3	17.1	16,314,000
**Cum10**	41.5	62.5	23.1	16,430,000
**E2SFCA**	45.3	60.2	33.1	17,936,000

^a^ Assuming one new physician per 10 underserved census tracts per year.

In the official PPR_Phys method, the analysis is performed per physician zone. These cover large areas, and therefore the zones where financial assistance is given or not are large contiguous areas (Figure 3). As mentioned earlier, 41.2% of all census tracts are underserved, which coincides with 51.9% of the total area and 35.3% of the total population of Belgium (Table 3). Assuming one new physician per 10 underserved census tracts per year, an amount of €16.3 million would be needed each year, which is the lowest amount of money of all selected methods.

When using the PPR_Mun method, and identifying shortage areas with the same criterion as in the official Impulseo I method but on the scale of municipalities, the ascription of financial assistance is now much more geographically diversified (Figure 4). Also, more census tracts are underserved (48.0%; see Table 3), resulting in a higher amount of money needed (almost €19 million). Approximately the same percentage of area is seen as shortage area (51.2%), but 47.7% of the population lives within these census tracts, which means that with this PPR_Mun method, census tracts with higher population densities are selected.

With the Dist3 method, the spatial distribution of census tracts where financial assistance should be given is striking. Here, shortage areas are mainly located outside city centres (Figure 4). The reason for this is the increasing distance to physicians outside city centres, because physicians are mainly located in city centres. From Table 3, this can also be deduced, because with the same percentage of census tracts as with the official PPR_Phys method (41.2%), an area of 66.3% and a population of only 17.1% is considered underserved.

It can be inferred from Figure 4 that with the Cum10 method, mainly physicians that settle in rural areas receive financial assistance. However, the geographical spread is much more clustered than with the Dist3 method and mainly in Wallonia physicians receive financial assistance. As with the previous method, a similar pattern is visible in Table 3: a large area of 60.2%, but only 23.1% of the population is underserved.

With the E2SFCA method, again a different spatial result is obtained (Figure 4). Now, mainly suburban and rural regions are underserved. With this method, 45.3% of census tracts are seen as shortage area, resulting in an amount of almost €18 million needed. Now, approximately the same percentage of population (33.1%), but a larger area (60.2%) is identified as underserved.

### Detailed spatial analysis

**Figure 5:**
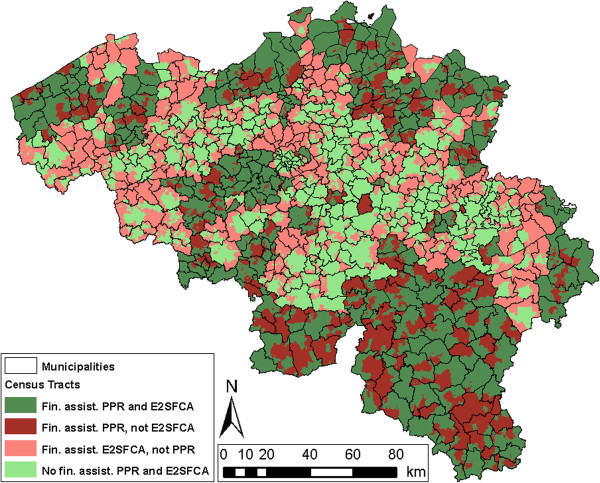
Detailed geographic analysis between the official Impulseo I method and the E2SFCA method, with the following four classes: ‘Financial assistance for PPR and E2SFCA’, ‘Financial assistance for PPR, but not for E2SFCA’, ‘Financial assistance for E2SFCA, but not for PPR’, and ‘No financial assistance for PPR and E2SFCA’.

In this section, the official Impulseo I method (PPR per physician zone) is geographically compared in more detail with the method that is conceptually most advanced and often used in recent studies: the E2SFCA method. Figure 5 shows all census tracts, divided in four classes, depending on whether or not the census tract is considered a shortage area in both methods.

The two classes represented in green (‘Financial assistance for PPR and E2SFCA’ and ‘No financial assistance for PPR and E2SFCA’) indicate the census tracts that are in both methods consistently classified as underserved/overserved. Underserved areas occur mainly in the periphery of the country, while overserved areas are mostly located in the central part of the country.

More important from a policy perspective is class ‘Financial assistance for PPR, but not for E2SFCA’. In the southern part of Belgium, many areas where physicians receive financial assistance by the official Impulseo I method would not have been identified as underserved on the basis of the E2SFCA method. Class ‘Financial assistance for E2SFCA, but not for PPR’ is also interesting for policy makers as these represent locations where currently no financial assistance is awarded while it might be appropriate. Mainly rural and suburban regions occur in this class.

## Discussion

### General discussion

Whether or not financial assistance should be awarded to physicians strongly depends on the selected method and spatial unit of analysis. Policy makers often define shortage areas by calculating PPR per physician zone, for the simple reason that it is an easy calculation and offers a readily understandable measure of accessibility. The advantage of this method is that it considers both the number of physicians and the population within the zone. However, it only offers a very crude representation of accessibility to primary health care because physician zones cover too large geographic areas [22,23,34]. Therefore, it cannot detect local variations in accessibility.

When calculating PPR per municipality, we observe slightly more underserved census tracts. This means that when using physician zones, some municipalities are not identified as shortage areas, while in fact they should be. There are however also some municipalities that are considered underserved, while they should not be. There can nevertheless be variations at an even smaller scale (e.g. census tracts), which cannot be detected using this method. Another disadvantage of this method is that interaction across borders is not sufficiently taken into account [10,14].

Other simple GIS methods (Dist1, Dist3, Cum5, and Cum10) are solely based on the supply (physicians), while the demand (population) is not accounted for. The results show that when using the Dist3 method, only few census tracts maintain their status as shortage area. The Cum10 method provides a result that coincides more with the official method, because both are based on the number of physicians.

FCA-based methods have the advantage of the small geographical scale of analysis at the level of census tracts, and taking interaction between population and physicians into account. From the FCA-based methods, the E2SFCA method is preferred because it accounts for distance decay by using a weight function [3,6]. The use of this method results in more shortage census tracts compared to the official Impulseo I method. However, only 51.6% of these census tracts were originally indicated as shortage areas. This means that 48.4% of all census tracts should be seen as shortage areas, while now they are not. When geographically comparing the results of the official Impulseo I method (PPR per physician zone) with the results of the E2SFCA method, the ascription of financial assistance is very different. Despite high population densities, urban areas are mostly not identified as shortage areas because of a dense concentration of physicians. Rural and suburban areas are often considered as shortage areas because physician accessibility is low. When using the official Impulseo I method, this pattern is less pronounced, because extreme values are filtered out. This aligns with the findings of Apparicio [16] and McGrail [21], who found that most accessibility problems occur in suburban areas, with low population density and mostly non-residential land use. Interestingly, however, the defined shortage areas follow the distribution of physicians much better when using the E2SFCA method.

The total number of census tracts where financial assistance should be awarded when a physician settles there is slightly higher with the E2SFCA method, so more money would be needed to invest in helping underserved areas. However, approximately the same population (33.1%) and a much bigger area (60.2%) is reached. Therefore, we would advice policy makers to use this method in future evaluations of accessibility to primary health care, because it aligns better with the actual distribution of physicians. In this way, and according the spatial analysis, the current policy in Belgium could be adjusted towards a more area-oriented approach.

**Figure 6:**
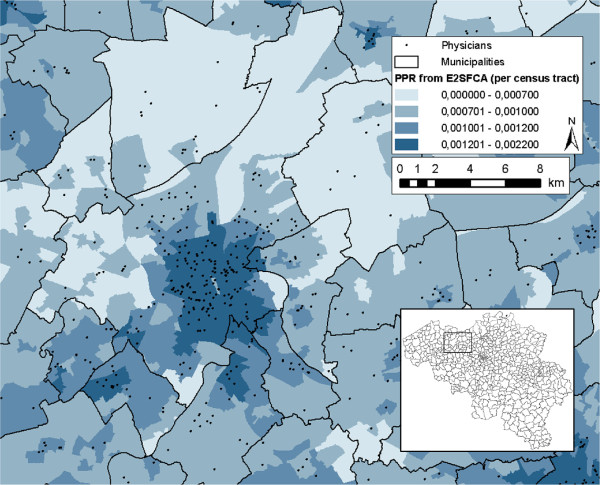
Choropleth map of the area around Ghent showing the PPR calculated with the E2SFCA method.

Additionally, we want to propose a different way of awarding financial assistance to physicians settling in shortage areas. Now, shortage areas are defined based on a sharp threshold (PPR <90 physicians/100,000 inhabitants). Alternatively, one could vary the financial award in function of the magnitude of shortage (see Figure 6 for an example). The higher the shortage, the higher the award a physician receives when settling there. Doing so, unequal accessibility to primary health care would possibly be conquered even more effectively, since more underserved areas would have a higher attraction to physicians.

### Study strengths and limitations

This study has several strengths. First, most previous studies using FCA-based methods use the centroid of the municipality where physicians live as physician location [2,3,14,27], whereas we use the exact location of physicians, leading to more accurate estimations of accessibility and reducing the influence of the MAUP.

Second, distance in this study has been considered following the street network, instead of following a straight line. In many studies (e.g. [2,16]) the lack of using street network data is considered a major limitation.

Third, the study area (Belgium) is larger and more populated relative to other applications of FCA-based methods in the context of accessibility to primary care. Our study area measures 30,528 km^2^ and has 10.8 million inhabitants, whereas in other studies the spatial coverage was limited to 19,774 km^2^ and 3.8 million inhabitants (nine counties in central Texas, USA; [25]), 14,331 km^2^ and 1.6 million (9 counties surrounding DeKalb in northern Illinois, USA; [2,3]), 4,258 km^2^ and 3.4 million (Montreal census metropolitan area, Canada; [16]), 499 km^2^ and 1.9 million (island of Montreal, Canada; [30]), and 177 km^2^ and 601,000 (Washington DC, USA; [14]). Two studies have bigger study areas, but a lower population: 230,000 km^2^ and 1.5 million inhabitants (rural Victoria, Australia; [34]), and 227,000 km^2^ and 5.5 million inhabitants (Victoria, Australia; [21]).

Fourth, the proposed study adds to the spatial coverage of evidence by spatially complementing existing studies that have been carried out primarily in North America (e.g. [2,3,14,16,25,30]) and Australia (e.g. [21,34]) with evidence from Europe.

Fifth, previous studies (ibid.) are all regional, while ours is nation-wide. A disadvantage of a regional study is that there can occur edge effects, because people can also go to a physician in a neighbouring region [2]. Our nation-wide study limits this, because it is less likely that inhabitants of Belgium will go to a doctor in a neighbouring country. Small edge effects can still occur within Belgium however. Belgium is separated in two regions with different languages, which implies that people prefer to go to a physician that speaks their native language. It was however difficult to control for this, because the language of physician and aggregated population was not known and there is a lot of bilingualism along the borders between the two regions. Also, with our nation-wide study, we can link our results with the conducted policy of the entire country to check whether the policy decisions correspond with the scientific results.

However, this study also has some limitations, most of which constitute interesting avenues for future work. First, accessibility is considered from the home location. However, people can also access primary health care from their working location, which can influence accessibility [35-37]. Nevertheless, in Belgium people shall probably be inclined to go to a physician in their residential neighbourhood whom they are familiar with, rather than searching for a physician near their work location.

Second, according to some studies, the size of the catchment should vary depending on whether it is urban or rural [3,29,34]. Despite the small differences between urban and rural populations in Belgium, adding a varying catchment size function (larger catchment sizes for rural populations) could improve the results.

Third, the population per census tract is now centered at its centroid. This is more accurate than looking at a scale level of a municipality or physician zone, but still is an approximation of reality. To improve this, one could consider each home location as a population location, from where accessibility is calculated. However, such data is often not available because of privacy issues and the calculation would be very computationally intensive.

Fourth, various socio-economic factors can also influence accessibility to primary health care [27]. Several studies have considered such factors as financial barriers, car-ownership, and educational level [10,38-42]. Also, data about the actual use of health services could provide information about revealed accessibility, instead of potential accessibility what is studied now. However, collecting this data is expensive [2], definitely at the scale of our study. Socio-economic attributes of physicians (e.g. ethnicity, gender, age) could also provide interesting information. This could however be incorporated in future research. Gender could be accounted for since the sex of a physician is known to be a barrier for certain population groups (e.g., young women [43]). Age could be dealt with because it will enable to identify and anticipate future shortage areas (i.e. areas that are likely to become underserved because of ageing physicians). Some other factors could also be incorporated in future research concerning this topic: e.g. the fact that physicians can also visit patients, visiting hours of physicians, average visit length which can vary per physicians, and congestion problems along the road network.

## Conclusions

Because of the simplicity of basic PPR methods, policy makers often use these to award financial assistance to shortage areas considering primary health care accessibility. Despite the fact that the PPR takes both supply and demand into consideration, a major disadvantage is its aggregated approach and the lack to detect local variations in accessibility, which arises because of local clustering and dispersion in the physician distribution.

Other GIS-based methods (e.g. distance to closest physician, cumulative opportunity, FCA-based methods) overcome this by not taking any boundaries into consideration. The E2SFCA method takes interaction between population and physicians into account, and considers distance decay by applying a weight function (which can be adjusted depending on the type or importance of a service). This method can however also be used to define accessibility to other services, e.g. dentists, post offices, hospitals, and schools. Network data is more and more accessible, and the effective use of network analysis software makes it possible to easily use more advanced GIS methods.

This manuscript has clearly shown that a different method and scale of analysis provides different results, not only in the total number of census tracts that are underserved, but also in the geographical spread. Currently, health policy makers often neglect the importance of these aspects in accessibility analyses. As a consequence, the distribution of financial incentives to prevent unequal spatial accessibility to primary health care may be biased.

## Competing interests

The authors declare that they have no competing interests.

## Authors’ contributions

BD carried out the main research and drafted the manuscript. TN participated in the study design and helped to draft the manuscript. NVdW and YDW coordinated the study. All authors reviewed the manuscript and approved the final version.

## Pre-publication history

The pre-publication history for this paper can be accessed here:

http://www.biomedcentral.com/1471-2296/14/122/prepub
